# Triglyceride-glucose index is associated with poor prognosis in acute coronary syndrome patients with prior coronary artery bypass grafting undergoing percutaneous coronary intervention

**DOI:** 10.1186/s12933-023-02029-6

**Published:** 2023-10-27

**Authors:** Shutong Dong, Zehao Zhao, Xin Huang, Meishi Ma, Zhiqiang Yang, Chu Fan, Hongya Han, Zhijian Wang, Dongmei Shi, Yujie Zhou

**Affiliations:** Department of Cardiology, Beijing Anzhen Hospital, Capital Medical University, Beijing Institute of Heart Lung and Blood Vessel Disease, Beijing Key Laboratory of Precision Medicine of Coronary Atherosclerotic Disease, Clinical center for coronary heart disease, Capital Medical University, Beijing, 100029 China

**Keywords:** Triglyceride-glucose index, Acute coronary syndrome, Coronary artery bypass grafting, Percutaneous coronary intervention

## Abstract

**Background:**

The triglyceride-glucose (TyG) index, which is a reliable substitute indicator for insulin resistance, has been considered an independent risk factor for long-term outcomes in patients with cardiovascular disease. However, it remains unknown whether the TyG index is associated with poor prognosis in acute coronary syndrome (ACS) patients with prior coronary artery bypass grafting (CABG) undergoing percutaneous coronary intervention (PCI).

**Methods:**

A total of 1158 ACS patients with prior CABG undergoing PCI were retrospectively studied. The TyG index was calculated by ln[fasting triglyceride (mg/dL) × fasting blood glucose (mg/dL)/2]. The primary endpoint was major adverse cardiovascular and cerebrovascular events (MACCE), a composite of all-cause death, nonfatal myocardial infarction, nonfatal stroke, and unplanned repeat revascularization.

**Results:**

During a median of 42-month follow-up, 350 patients (30.2%) experienced at least one endpoint event. Based on the optimal cut-off value of the TyG index, patients were divided into the high TyG index group and the low TyG index group. Patients in the high TyG index group had higher risks of MACCE (35.3% vs. 25.3%, p < 0.001), major adverse cardiovascular events (MACE) (31.1% vs. 23.4%, p = 0.003), nonfatal stroke (4.2% vs. 1.9%, p = 0.022) and unplanned repeat revascularization (19.4% vs. 11.3%, p < 0.001) than those in the low TyG index group. Cox regression analysis demonstrated that there was an independent association between the TyG index and MACCE regardless of whether the TyG index was a continuous or categorical variable (HR 1.42, 95% CI 1.09–1.86, p = 0.009; HR 1.53, 95% CI 1.16–2.01, p = 0.003, respectively). Restricted cubic spline curve exhibited that the relationship between the TyG index and MACCE was linear (p for non-linear = 0.595, p for overall = 0.005). By incorporating the TyG index groups into baseline risk model, the accuracy of predicting MACCE was improved [AUC: baseline risk model, 0.618 vs. baseline risk model + TyG index groups, 0.636, p for comparison = 0.042].

**Conclusions:**

The TyG index is independently associated with MACCE, suggesting that the TyG index may serve as a valid indicator for predicting poor prognosis in ACS patients with prior CABG undergoing PCI.

**Supplementary Information:**

The online version contains supplementary material available at 10.1186/s12933-023-02029-6.

## Background

Coronary artery disease (CAD) remains the leading cause of morbidity and mortality, and a major burden on global healthcare systems [1, 2]. Currently, the revascularization strategies utilized in CAD include coronary artery bypass grafting (CABG) and percutaneous coronary intervention (PCI). Many patients received CABG, especially those with severe left main coronary artery (LM) disease or multivessel disease [3]. Patients with prior CABG often need repeated revascularization because of recurrent angina or acute coronary syndrome (ACS), which is attributed to the high rate of graft failure and the rapid progression of atherosclerosis in both native and graft vessels [4, 5]. Based on current studies and guidelines, PCI is recommended as the preferred method for repeat revascularization in such patients rather than repeat CABG [3, 6]. Although the PCI was successful, there were still poor long-term outcomes in patients with prior CABG undergoing PCI. Therefore, it is urgent to explore new predictors to identify high-risk patients and take earlier interventions to reduce the incidence of adverse cardiovascular (CV) events and improve prognosis.

Insulin resistance (IR) is characterized by decreased sensitivity and responsiveness of insulin target organs or tissues to insulin, which impairs the abilities of glucose uptake and utilization, leading to hyperinsulinemia [7]. IR plays an important role in the pathogenesis of type 2 diabetes mellitus (T2DM) and metabolic syndrome (MetS), and has a strong association with the incidence and development of cardiovascular disease (CVD) [8, 9]. Although the hyperinsulinaemic-euglycaemic clamp (HEC) is the gold standard for assessing IR and can directly measure insulin sensitivity, it is rarely applied in clinical practice due to its complicated, time-consuming, expensive and invasive procedures. In addition, homeostasis model assessment of IR (HOMA-IR) is widely used as an indirect method for evaluating IR, but it is limited in clinical practice because of a lack of standard insulin measurements and the vulnerability to interference from various factors.

The triglyceride-glucose (TyG) index is derived from fasting blood glucose (FBG) and fasting triglyceride (TG). It is a simple, reliable, and readily available surrogate, which shows its superiority in IR evaluation [10]. There was a close correlation between the TyG index and HEC or HOMA-IR, and even the TyG index was more effective than HOMA-IR in some conditions [11–14]. Previous studies revealed that the TyG index was not only significantly associated with MetS, hypertension, T2DM, and atherosclerosis [15–18], but also an independent risk factor for the occurrence and development of CVD and poor prognosis [19, 20]. Furthermore, the TyG index has also been confirmed to be significantly associated with poor prognosis in ACS patients who underwent PCI, regardless of the presence or absence of T2DM [21, 22].

However, none of the studies evaluated the prognostic significance of the TyG index in ACS patients with prior CABG undergoing PCI. Our study included ACS patients with prior CABG undergoing PCI, filling the gap in such a high-risk population. Therefore, the aims of the present study were to explore the predictive valve of the TyG index for adverse CV events in the above patients, and further determine the ability of the TyG index in risk stratification.

## Methods

### Study population

**Fig. 1:**
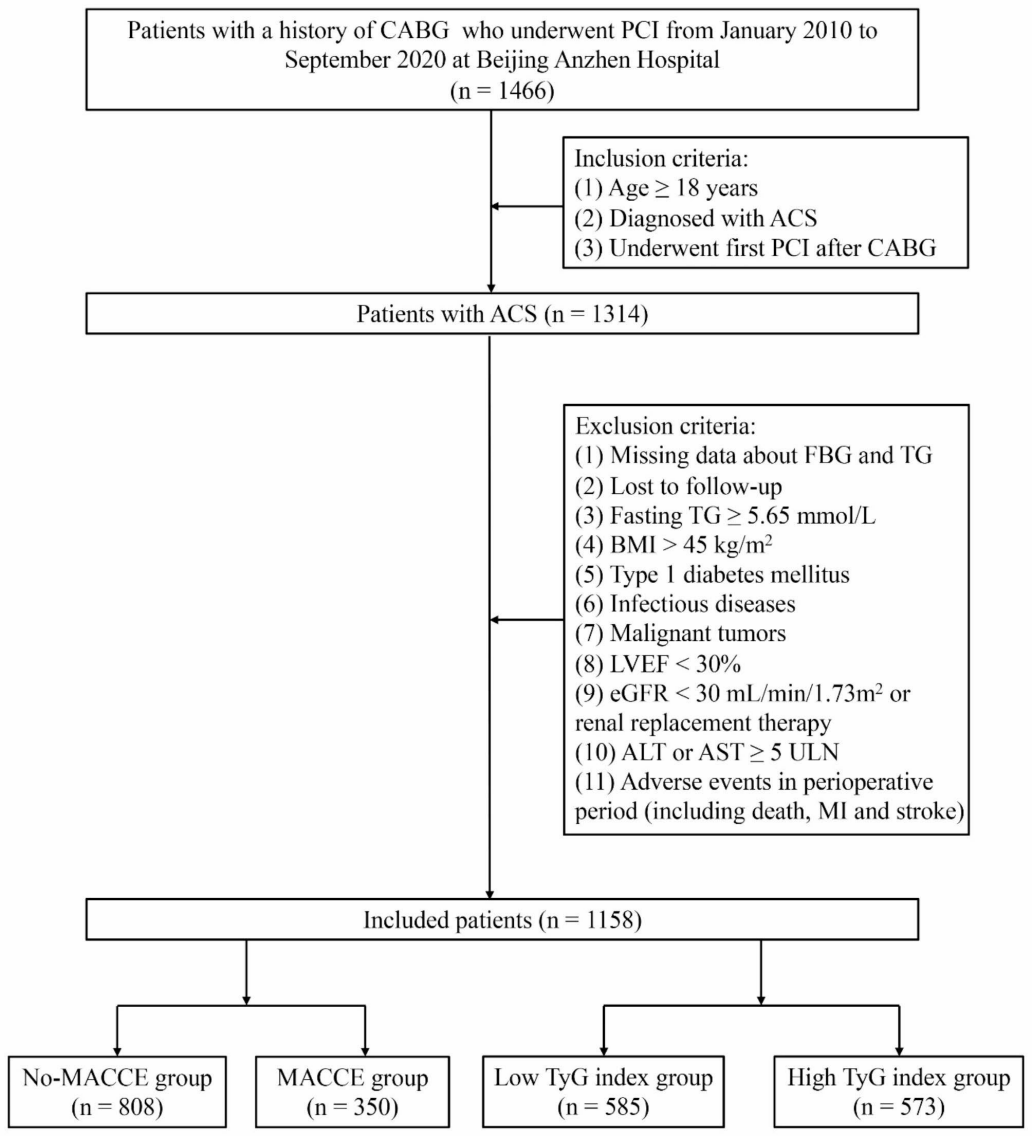
Flow chart for the enrollment of study population. CABG, coronary artery bypass grafting; PCI, percutaneous coronary intervention; ACS, acute coronary syndrome; FBG, fasting blood glucose; TG, triglyceride; BMI, body mass index; LVEF, left ventricular ejection fraction; eGFR, estimated glomerular filtration rate; ALT, alanine transaminase; AST, aspartate transaminase; ULN, upper limit of normal; MI, myocardial infarction; MACCE, major adverse cardiavascular and cerebrovascular events; TyG, triglyceride-glucose

This is a single-center, retrospective, and observational study that consecutively included 1466 patients with a history of CABG who underwent PCI from January 2010 to September 2020 at Beijing Anzhen Hospital. The inclusion criteria were as follows: (1) age ≥ 18 years; (2) patients diagnosed with ACS; (3) Patients underwent first PCI after CABG. The exclusion criteria included missing FBG and TG data, loss to follow-up (Fig. 1). In the final analysis, 1158 patients were ultimately included. All patients were stratified by the occurrence of major adverse cardiovascular and cerebrovascular events (MACCE) during the follow-up period into the no-MACCE group (n = 808) and the MACCE group (n = 350). Moreover, all patients were further classified into the low TyG index group (n = 585) and the high index group (n = 573) based on the optimal cut-off value of the TyG index. The Declaration of Helsinki was strictly followed in the study protocol. With the approval of the Clinical Research Ethics Committee of Beijing Anzhen Hospital, informed consent was waived due to the retrospective nature of this study. All personal information regarding patient identity was removed.

### Data collection and definitions

Demographics, vital signs, current smoking, family history of CAD, medical histories, clinical diagnosis, laboratory measurements, echocardiography reports, angiography results, PCI outcomes, and medications at discharge were collected from Beijing Anzhen Hospital’s electronic medical record system. Weight (kg)/[height (m)]^2^ was used to calculate body mass index (BMI). Current smoking was defined as quitting smoking for less than one year or continuing smoking on admission. ACS, including unstable angina (UA), non-ST-segment elevation myocardial infarction (NSTEMI), and ST-segment elevation myocardial infarction (STEMI), was defined according to current guidelines [23, 24]. Laboratory measurements were determined by standard techniques in the central laboratory of Beijing Anzhen Hospital. Peripheral venous blood samples were extracted after fasting for more than 12 h before PCI. The TyG index was calculated using the formula ln(TG [mg/dL] × FBG [mg/dL]/2). All patients were treated with coronary angiography and PCI following standard procedures [25]. The PCI outcomes were interpreted and recorded by no less than two professional and independent cardiologists.

### Follow-up and endpoint

All patients were followed up by professionally trained personnel through telephone or outpatient clinics. Follow-up continued until April 2022, unless withdrawal or death occurred. The primary study endpoint was a composite of MACCE, which consisted of all-cause death, nonfatal stroke, nonfatal myocardial infarction (MI), and unplanned repeat revascularization. The secondary endpoints were each individual component of MACCE and major adverse cardiovascular events (MACE) (a composite of all-cause death, nonfatal MI, and unplanned repeat revascularization). Nonfatal MI was defined as the typical symptoms of myocardial ischemia accompanied by elevated biomarkers of myocardial injury and/or dynamic changes in electrocardiograms. Nonfatal stroke included cerebral infarction and cerebral hemorrhage diagnosed according to imaging examination. Unplanned repeat revascularization was defined as ischemia-driven revascularization. The most severe event was seen as the endpoint event if more than one event occurred during the follow-up (all-cause death > nonfatal stroke > nonfatal MI > unplanned repeat revascularization). The first event after PCI was selected if the endpoint events occurred more than once. All endpoint events were adjudicated independently by at least two experienced cardiologists.

### Statistical analysis

The mean ± standard deviation (SD) or medians with interquartile ranges (IQR) were used to describe continuous variables, and comparisons between the groups were conducted through either the independent sample t-test or Wilcoxon rank sum test. The representation of categorical variables was in the form of frequencies (percentages), and comparisons between the groups were performed using either Pearson’s chi-squared test or Fisher’s exact test. Correlation analysis between the TyG index and cardiovascular risk factors was conducted using either the Pearson correlation test or Spearman’s rank sum test. Receiver operating characteristic (ROC) curve analysis was used to identify the optimal cut-off value of the TyG index for predicting MACCE, and further evaluate the incremental effect of TyG index groups on discrimination capacity beyond the baseline risk model. DeLong’s test was employed to obtain and compare the area under the curve (AUC). Kaplan-Meier survival analysis was performed to evaluate the occurrence of endpoint events between the two groups, and compared by the log-rank test. To verify whether the TyG index could serve as an independent predictor of the occurrence of MACCE, univariate and multivariate Cox regression analyses were performed with the results displayed as hazard ratio (HR) and 95% confidence interval (CI). Variables with significant differences in the univariate analysis or clinical significance were included in the multivariate Cox regression analysis. Additionally, we developed four different Cox proportional hazards models as described below: Model 1 was adjusted for age, male, BMI, systolic blood pressure (SBP), current smoking, family history of CAD, hypertension, T2DM, dyslipidemia, prior MI, prior PCI, prior stroke, peripheral artery disease (PAD), heart failure (HF), and chronic kidney disease (CKD); Model 2 was adjusted for variables of model 1 and clinical diagnosis, hemoglobin (Hb), high-sensitivity C-reactive protein (hs-CRP), estimated glomerular filtration rate (eGFR), total cholesterol (TC), low-density lipoprotein cholesterol (LDL-C), high-density lipoprotein cholesterol (HDL-C), glycosylated hemoglobin A1c (HbA1c), and left ventricular ejection fraction (LVEF); Model 3 was adjusted for variables of model 2 and dual antiplatelet therapy (DAPT), angiotensin-converting enzyme inhibitor or angiotensin receptor blocker (ACEI/ARB), angiotensin receptor-neprilysin inhibitor (ARNI), antidiabetic agents, and statins at discharge; Model 4 was adjusted for the variables of model 3 and LM disease, multivessel disease, chronic total occlusion (CTO), in-stent restenosis, thrombotic disease, target vessel selection, percutaneous transluminal coronary angioplasty (PTCA), number of stents, interval time from CABG to PCI, and PCI success. In Cox regression analysis, the TyG index was presented as a continuous or a categorical variable to validate the independent risk factors for MACCE in each model. Besides, restricted cubic spline (RCS) curve was constructed to illustrate the linear or non-linear relationship between the TyG index and MACCE with the adjustment of Model 4. Subgroup analysis was further performed to investigate whether the predictive value of the TyG index for MACCE was consistent across subgroups. Moreover, the net reclassification improvement (NRI) and integrated discrimination improvement (IDI) were tested to explore the discrimination capacity of the TyG index for predicting MACCE.

Statistical analyses were conducted using SPSS 26.0 (IBM Corporation, IL, USA) and R Programming Language 4.2.1 (Vienna, Austria). All p values were two-tailed, and statistical significance was defined by the p value < 0.05.

## Results

### Baseline characteristics

In the present study, a total of 1158 patients were ultimately included. The mean age was 64.5 ± 8.0 years, and 872 (75.3%) patients were male. The baseline characteristics of the total population stratified by MACCE are shown in Table 1. Patients in the MACCE group had a higher TyG index compared with those in the no-MACCE group (9.08 ± 0.64 vs. 8.94 ± 0.60, p < 0.001). Besides, patients in the MACCE group had significantly higher levels of BMI, SBP, hs-CRP, creatinine, TC, LDL-C, and FBG, but lower levels of Hb than those in the no-MACCE group. Compared with the no-MACCE group, there were more patients who underwent graft vessel PCI and fewer patients who underwent native vessel PCI in the MACCE group. Meanwhile, there was a significant difference in the selection of target vessel (only native vessel, only graft vessel, both native and graft vessels) between the two groups. Additionally, the proportion of ACEI/ARB medication at discharge was significantly higher in the MACCE group than those in the no-MACCE group. Other characteristics between the two groups were not significantly different.

**Table 1:** Baseline characteristics of population stratified by MACCE

Characteristics	Total population (N = 1,158)	No-MACCE (N = 808)	MACCE (N = 350)	p value
**Demographics**				
Age (years)	64.5 ± 8.0	64.3 ± 8.0	64.7 ± 7.8	0.445
Male, n (%)	872 (75.3%)	618 (76.5%)	254 (72.6%)	0.156
BMI (kg/m^2^)	26.19 ± 3.19	26.02 ± 3.04	26.59 ± 3.49	0.008
SBP (mmHg)	130 ± 17	129 ± 16	132 ± 18	0.031
DBP (mmHg)	75 ± 11	74 ± 10	76 ± 11	0.060
Heart rate (bpm)	67 ± 10	67 ± 10	68 ± 11	0.069
Current smoking, n (%)	273 (23.6%)	182 (22.5%)	91 (26.0%)	0.201
Family history of CAD, n (%)	106 (9.2%)	68 (8.4%)	38 (10.9%)	0.186
**Medical histories**				
Hypertension, n (%)	837 (72.3%)	578 (71.5%)	259 (74.0%)	0.389
Dyslipidemia, n (%)	1,152 (99.5%)	805 (99.6%)	347 (99.1%)	0.374
T2DM, n (%)	533 (46.0%)	358 (44.3%)	175 (50.0%)	0.074
Prior MI, n (%)	557 (48.1%)	377 (46.7%)	180 (51.4%)	0.136
Prior PCI, n (%)	249 (21.5%)	168 (20.8%)	81 (23.1%)	0.371
Prior stroke, n (%)	147 (12.7%)	103 (12.7%)	44 (12.6%)	0.934
PAD, n (%)	111 (9.6%)	76 (9.4%)	35 (10.0%)	0.752
HF, n (%)	74 (6.4%)	52 (6.4%)	22 (6.3%)	0.924
CKD, n (%)	38 (3.3%)	22 (2.7%)	16 (4.6%)	0.105
**Clinical diagnosis**				0.446
UA, n (%)	1,008 (87.0%)	710 (87.9%)	298 (85.1%)	
NSTEMI, n (%)	115 (9.9%)	75 (9.3%)	40 (11.4%)	
STEMI, n (%)	35 (3.0%)	23 (2.8%)	12 (3.4%)	
**Laboratory measurements**				
WBC (x10^9^/L)	6.82 ± 1.72	6.80 ± 1.69	6.86 ± 1.79	0.549
Hb (g/L)	141.69 ± 15.81	142.52 ± 15.34	139.77 ± 16.70	0.009
PLT (x10^9^/L)	204.72 ± 56.08	204.66 ± 54.73	204.87 ± 59.15	0.955
hs-CRP (mg/L)	1.24 (0.55, 3.23)	1.08 (0.51, 2.77)	1.81 (0.69, 4.04)	< 0.001
Creatinine (𝝁mmol/L)	77.52 ± 19.95	76.56 ± 18.04	79.75 ± 23.64	0.024
eGFR (mL/min/1.73m^2^)	90.43 ± 22.60	91.09 ± 21.51	88.91 ± 24.90	0.155
TG (mmol/L)	1.66 ± 0.81	1.63 ± 0.80	1.72 ± 0.85	0.088
TC (mmol/L)	3.99 ± 1.01	3.92 ± 0.97	4.16 ± 1.06	< 0.001
LDL-C (mmol/L)	2.36 ± 0.86	2.30 ± 0.84	2.49 ± 0.90	< 0.001
HDL-C (mmol/L)	1.01 ± 0.23	1.01 ± 0.23	1.01 ± 0.24	0.639
FBG (mmol/L)	7.14 ± 3.01	6.92 ± 2.90	7.65 ± 3.19	< 0.001
HbA1c (%)	6.76 ± 1.34	6.74 ± 1.33	6.80 ± 1.36	0.544
TyG index	8.98 ± 0.61	8.94 ± 0.60	9.08 ± 0.64	< 0.001
LVEF (%)	59 ± 8	59 ± 8	58 ± 8	0.311
**GRACE risk score**	99 ± 34	99 ± 33	100 ± 36	0.518
**Angiographic results**				
LM disease, n (%)	358 (30.9%)	253 (31.3%)	105 (30.0%)	0.713
Multivessel disease, n (%)	1,059 (94.8%)	739 (94.4%)	320 (95.8%)	0.325
Number of LIMA, n (%)	1 (1, 1)	1 (1, 1)	1 (1, 1)	0.233
Number of SVG, n (%)	2 (1, 3)	2 (1, 3)	2 (1, 3)	0.584
CTO, n (%)	1,058 (91.5%)	737 (91.2%)	321 (92.2%)	0.565
Thrombotic disease, n (%)	35 (3.0%)	24 (3.0%)	11 (3.1%)	0.875
In-stent restenosis, n (%)	101 (8.7%)	76 (9.4%)	25 (7.2%)	0.215
**PCI outcomes**				
Target vessel territory				
Native vessel, n (%)	1,038 (89.6%)	738 (91.3%)	300 (85.7%)	0.004
LM, n (%)	141 (12.2%)	108 (13.4%)	33 (9.4%)	
LAD, n (%)	284 (24.5%)	205 (25.4%)	79 (22.6%)	
LCX, n (%)	389 (33.6%)	263 (32.5%)	126 (36.0%)	
RCA, n (%)	549 (47.4%)	390 (48.3%)	159 (45.4%)	
Graft vessel, n (%)	182 (15.7%)	115 (14.2%)	67 (19.1%)	0.035
LIMA, n (%)	4 (0.3%)	3 (0.4%)	1 (0.3%)	
SVG, n (%)	178 (15.4%)	112 (13.9%)	66 (18.9%)	
Target vessel selection, n (%)				0.015
Only native vessel	976 (84.3%)	693 (85.8%)	283 (80.9%)	
Only graft vessel	120 (10.4%)	70 (8.7%)	50 (14.3%)	
Both native and graft vessels	62 (5.4%)	45 (5.6%)	17 (4.9%)	
PTCA, n (%)	114 (9.8%)	77 (9.5%)	37 (10.6%)	0.585
DCB application, n (%)	41 (3.5%)	33 (4.1%)	8 (2.3%)	0.128
BMS implantation, n (%)	2 (0.2%)	1 (0.1%)	1 (0.3%)	0.513
DES implantation, n (%)	1,027 (99.8%)	728 (99.9%)	299 (99.7%)	0.498
Number of treated lesions	1 (1, 2)	1 (1, 2)	1 (1, 2)	0.235
Number of stents	1 (1, 2)	1 (1, 2)	1 (1, 2)	0.487
Interval time from CABG to PCI (years)	6.3 ± 4.4	6.2 ± 4.3	6.5 ± 4.5	0.220
PCI success, n (%)	1,024 (88.4%)	724 (89.6%)	300 (85.7%)	0.057
**Medications at discharge**				
DAPT, n (%)	1,140 (98.4%)	793 (98.1%)	347 (99.1%)	0.207
ACEI/ARB, n (%)	592 (51.1%)	392 (48.5%)	200 (57.1%)	0.007
ARNI, n (%)	12 (1.0%)	8 (1.0%)	4 (1.1%)	0.761
𝜷-Blocker, n (%)	968 (83.6%)	681 (84.3%)	287 (82.0%)	0.336
Statins, n (%)	1,146 (99.0%)	802 (99.3%)	344 (98.3%)	0.202
Antidiabetic agents, n (%)	483 (41.7%)	323 (40.0%)	160 (45.7%)	0.069

MACCE, major adverse cardiavascular and cerebrovascular events; BMI, body mass index; SBP, systolic blood pressure; DBP, diastolic blood pressure; CAD, coronary artery disease; T2DM, type 2 diabetes mellitus; MI, myocardial infarction; PCI, percutaneous coronary intervention; PAD, peripheral artery disease; HF, heart failure; CKD, chronic kidney disease; UA, unstable angina; NSTEMI, non‑ST‑segment elevation myocardial infarction; STEMI, ST‑segment elevation myocardial infarction; WBC, white blood cell; Hb, hemoglobin; PLT, platelet; hs-CRP, high-sensitivity C-reactive protein; eGFR, estimated glomerular filtration rate; TG, triglyceride; TC, total cholesterol; LDL-C, low-density lipoprotein cholesterol; HDL-C, high-density lipoprotein cholesterol; FBG, fasting blood glucose; HbA1c, glycosylated hemoglobin A1c; TyG, triglycerid-glucose; LVEF, left ventricular ejection fraction; GRACE, global registry of acute coronary events; LM, left main coronary artery; LIMA, left internal mammary artery; SVG, saphenous vein graft; CTO, chronic total occlusion; LAD, left anterior descending artery; LCX, left circumflex artery; RCA, right coronary artery; PTCA, percutaneous transluminal coronary angioplasty; DCB, drug-coated balloon; BMS, bare mental stent; DES, drug-eluting stent; CABG, coronary artery bypass grafting; DAPT, dual antiplatelet therapy; ACEI/ARB, angiotensin converting enzyme inhibitor or angiotensin receptor blocker; ARNI, angiotensin receptor-neprilysin inhibitor

**Fig. 2:**
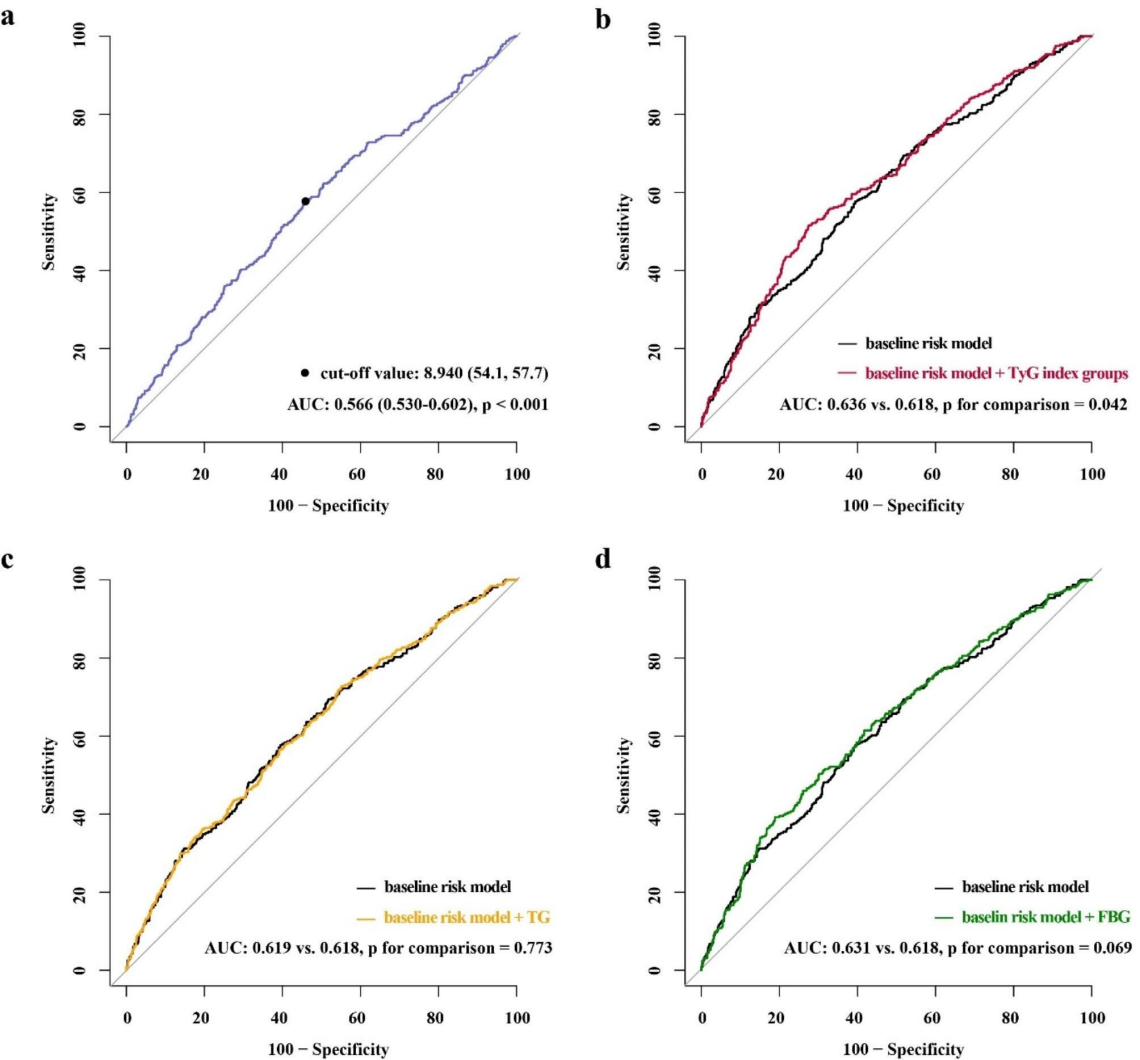
ROC curves evaluating the diagnostic performance of TyG index and its components for MACCE. **a** ROC curve analysis of TyG index for MACCE; **b** baseline risk model vs. + TyG index groups; **c** baseline risk model vs. + TG; **d** baseline risk model vs. + FBG. The baseline risk model includes age, male, BMI, SBP, current smoking, family history of CAD, hypertension, dyslipidemia, T2DM, prior MI, prior PCI, prior stroke, PAD, clinical diagnosis, eGFR, LVEF, DAPT, ACEI/ARB, antidiabetic agents, statins at discharge, LM disease, multivessel disease, CTO, in-stent restenosis, target vessel selection, number of LIMA, number of SVG, number of treated lesions, number of stents. AUC, area under the curve; TyG, triglyceride-glucose; TG, triglyceride; FBG, fasting blood glucose; other abbreviations as in Table 1

The AUC of the TyG index for predicting MACCE was 0.566 (95% CI 0.530–0.602, p < 0.001) in ROC curve analysis. With a sensitivity of 57.7% and a specificity of 45.9%, the optimal cut-off value of the TyG index for predicting MACCE was 8.94 (Fig. 2a). The baseline characteristics of the total population stratified by the optimal cut-off value of the TyG index are shown in Table 2. In comparison to the low TyG index group, patients were more prone to be younger and female, and had higher proportions of family history of CAD, T2DM, and prior PCI in the high TyG index group. Besides, the levels of BMI, heart rate, white blood cell (WBC), platelet (PLT), hs-CRP, TG, TC, LDL-C, FBG, and HbA1c in the high TyG index group were significantly higher, but the levels of eGFR and HDL-C were significantly lower. Furthermore, the interval time from CABG to PCI was longer in patients with a higher TyG index, and there were more patients in the high TyG index group treated with ACEI/ARB medication and antidiabetic agents at discharge. Other characteristics between the two groups without significant difference.

**Table 2:** Baseline characteristics of population stratified by the optimal cut-off value of TyG index

Characteristics	Total population (N = 1,158)	Low TyG index group (N = 585)	High TyG index group (N = 573)	p value
**Demographics**				
Age (years)	64.5 ± 8.0	65.1 ± 8.0	63.8 ± 7.9	0.005
Male, n (%)	872 (75.3%)	468 (80.0%)	404 (70.5%)	< 0.001
BMI (kg/m^2^)	26.19 ± 3.19	25.90 ± 3.22	26.49 ± 3.13	0.002
SBP (mmHg)	130 ± 17	129 ± 17	131 ± 17	0.101
DBP (mmHg)	75 ± 11	75 ± 11	75 ± 10	0.844
Heart rate (bpm)	67 ± 10	67 ± 10	68 ± 10	0.006
Current smoking, n (%)	273 (23.6%)	139 (23.8%)	134 (23.4%)	0.881
Family history of CAD, n (%)	106 (9.2%)	42 (7.2%)	64 (11.2%)	0.019
**Medical histories**				
Hypertension, n (%)	837 (72.3%)	411 (70.3%)	426 (74.3%)	0.120
Dyslipidemia, n (%)	1,152 (99.5%)	583 (99.7%)	569 (99.3%)	0.447
T2DM, n (%)	533 (46.0%)	201 (34.4%)	332 (57.9%)	< 0.001
Prior MI, n (%)	557 (48.1%)	284 (48.5%)	273 (47.6%)	0.758
Prior PCI, n (%)	249 (21.5%)	110 (18.8%)	139 (24.3%)	0.024
Prior stroke, n (%)	147 (12.7%)	83 (14.2%)	64 (11.2%)	0.123
PAD, n (%)	111 (9.6%)	57 (9.7%)	54 (9.4%)	0.854
HF, n (%)	74 (6.4%)	38 (6.5%)	36 (6.3%)	0.882
CKD, n (%)	38 (3.3%)	18 (3.1%)	20 (3.5%)	0.693
**Clinical diagnosis**				0.087
UA, n (%)	1,008 (87.0%)	521 (89.1%)	487 (85.0%)	
NSTEMI, n (%)	115 (9.9%)	47 (8.0%)	68 (11.9%)	
STEMI, n (%)	35 (3.0%)	17 (2.9%)	18 (3.1%)	
**Laboratory measurements**				
WBC (x10^9^/L)	6.82 ± 1.72	6.62 ± 1.72	7.02 ± 1.70	< 0.001
Hb (g/L)	141.69 ± 15.81	141.62 ± 16.22	141.76 ± 15.39	0.874
PLT (x10^9^/L)	204.72 ± 56.08	194.82 ± 50.93	214.83 ± 59.24	< 0.001
hs-CRP (mg/L)	1.24 (0.55, 3.23)	0.96 (0.45, 2.86)	1.60 (0.71, 3.39)	< 0.001
Creatinine (𝝁mmol/L)	77.52 ± 19.95	76.87 ± 19.51	78.18 ± 20.37	0.264
eGFR (mL/min/1.73m^2^)	90.43 ± 22.60	91.75 ± 22.88	89.08 ± 22.25	0.044
TG (mmol/L)	1.66 ± 0.81	1.14 ± 0.33	2.18 ± 0.82	< 0.001
TC (mmol/L)	3.99 ± 1.01	3.74 ± 0.95	4.25 ± 1.00	< 0.001
LDL-C (mmol/L)	2.36 ± 0.86	2.22 ± 0.84	2.50 ± 0.86	< 0.001
HDL-C (mmol/L)	1.01 ± 0.23	1.06 ± 0.24	0.97 ± 0.21	< 0.001
FBG (mmol/L)	7.14 ± 3.01	5.78 ± 1.36	8.53 ± 3.55	< 0.001
HbA1c (%)	6.76 ± 1.34	6.35 ± 1.00	7.18 ± 1.50	< 0.001
TyG index	8.98 ± 0.61	8.50 ± 0.32	9.47 ± 0.43	< 0.001
LVEF (%)	59 ± 8	59 ± 9	58 ± 8	0.589
**GRACE risk score**	99 ± 34	99 ± 32	99 ± 35	0.935
**Angiographic results**				
LM disease, n (%)	358 (30.9%)	191 (32.6%)	167 (29.1%)	0.197
Multivessel disease, n (%)	1,059 (94.8%)	534 (94.2%)	525 (95.5%)	0.337
Number of LIMA, n (%)	1 (1, 1)	1 (1, 1)	1 (1, 1)	0.226
Number of SVG, n (%)	2 (1, 3)	2 (1, 3)	2 (1, 3)	0.892
CTO, n (%)	1,058 (91.5%)	532 (91.1%)	526 (92.0%)	0.599
Thrombotic disease, n (%)	35 (3.0%)	15 (2.6%)	20 (3.5%)	0.357
In-stent restenosis, n (%)	101 (8.7%)	44 (7.5%)	57 (9.9%)	0.146
**PCI outcomes**				
Target vessel territory				
Native vessel, n (%)	1,038 (89.6%)	530 (90.6%)	508 (88.7%)	0.278
LM, n (%)	141 (12.2%)	75 (12.8%)	66 (11.5%)	
LAD, n (%)	284 (24.5%)	166 (28.4%)	118 (20.6%)	
LCX, n (%)	389 (33.6%)	180 (30.8%)	209 (36.5%)	
RCA, n (%)	549 (47.4%)	282 (48.2%)	267 (46.6%)	
Graft vessel, n (%)	182 (15.7%)	89 (15.2%)	93 (16.2%)	0.635
LIMA, n (%)	4 (0.3%)	3 (0.5%)	1 (0.2%)	
SVG, n (%)	178 (15.4%)	86 (14.7%)	92 (16.1%)	
Target vessel selection, n (%)				0.460
Only native vessel	976 (84.3%)	496 (84.8%)	480 (83.8%)	
Only graft vessel	120 (10.4%)	55 (9.4%)	65 (11.3%)	
Both native and graft vessels	62 (5.4%)	34 (5.8%)	28 (4.9%)	
PTCA, n (%)	114 (9.8%)	55 (9.4%)	59 (10.3%)	0.609
DCB application, n (%)	41 (3.5%)	22 (3.8%)	19 (3.3%)	0.682
BMS implantation, n (%)	2 (0.2%)	0 (0.0%)	2 (0.3%)	0.245
DES implantation, n (%)	1,027 (99.8%)	518 (100.0%)	509 (99.6%)	0.246
Number of treated lesions	1 (1, 2)	1 (1, 2)	1 (1, 2)	0.873
Number of stents	1 (1, 2)	1 (1, 2)	2 (1, 2)	0.322
Interval time from CABG to PCI (years)	6.3 ± 4.4	6.0 ± 4.3	6.6 ± 4.4	0.023
PCI success, n (%)	1,024 (88.4%)	526 (89.9%)	498 (86.9%)	0.110
**Medications at discharge**				
DAPT, n (%)	1,140 (98.4%)	578 (98.8%)	562 (98.1%)	0.320
ACEI/ARB, n (%)	592 (51.1%)	282 (48.2%)	310 (54.1%)	0.045
ARNI, n (%)	12 (1.0%)	5 (0.9%)	7 (1.2%)	0.538
𝜷-Blocker, n (%)	968 (83.6%)	481 (82.2%)	487 (85.0%)	0.203
Statins, n (%)	1,146 (99.0%)	580 (99.1%)	566 (98.8%)	0.538
Antidiabetic agents, n (%)	483 (41.7%)	179 (30.6%)	304 (53.1%)	< 0.001

All abbreviations as in Table 1

### Relationship between the TyG index and cardiovascular risk factors

The TyG index was related to various cardiovascular risk factors according to correlation analysis. As shown in additional file 1: Table S1, the TyG index had a positive association with BMI, T2DM, hs-CRP, TC, LDL-C, and HbA1c, while a negative association with age, male, and HDL-C (all p < 0.001).

### Predictive value of the TyG index for endpoint events

During a median follow-up of 42 months (IQR, 24–65 months), 350 (30.2%) patients developed MACCE, which included 88 (7.6%) all-cause death, 50 (4.3%) nonfatal MI, 35 (3.0%) nonfatal stroke, and 177 (15.3%) unplanned repeat revascularization. Compared with the low TyG index group, the rates of MACCE (35.3% vs. 25.3%, p < 0.001), MACE (31.1% vs. 23.4%, p = 0.003), nonfatal stroke (4.2% vs. 1.9%, p = 0.022), and unplanned repeat revascularization (19.4% vs. 11.3%, p < 0.001) were significantly higher in the high TyG index group. However, the rates of all-cause death and nonfatal MI were similar between the two groups (Table 3).

**Table 3:** Comparison of endpoint events stratified by the optimal cut-off value of TyG index

Variable, n (%)	Total population	Low TyG index group	High TyG index group	p value
	(N = 1,158)	(N = 585)	(N = 573)	
MACCE	350 (30.2%)	148 (25.3%)	202 (35.3%)	< 0.001
MACE	315 (27.2%)	137 (23.4%)	178 (31.1%)	0.003
All-cause death	88 (7.6%)	45 (7.7%)	43 (7.5%)	0.904
Non-fatal stroke	35 (3.0%)	11 (1.9%)	24 (4.2%)	0.022
Non-fatal MI	50 (4.3%)	26 (4.4%)	24 (4.2%)	0.830
Unplanned repeat revascularization	177 (15.3%)	66 (11.3%)	111 (19.4%)	< 0.001

TyG, triglyceride-glucose; MACCE, major adverse cardiavascular and cerebrovascular events; MACE, major adverse cardiavascular events; MI, myocardial infarction

**Fig. 3:**
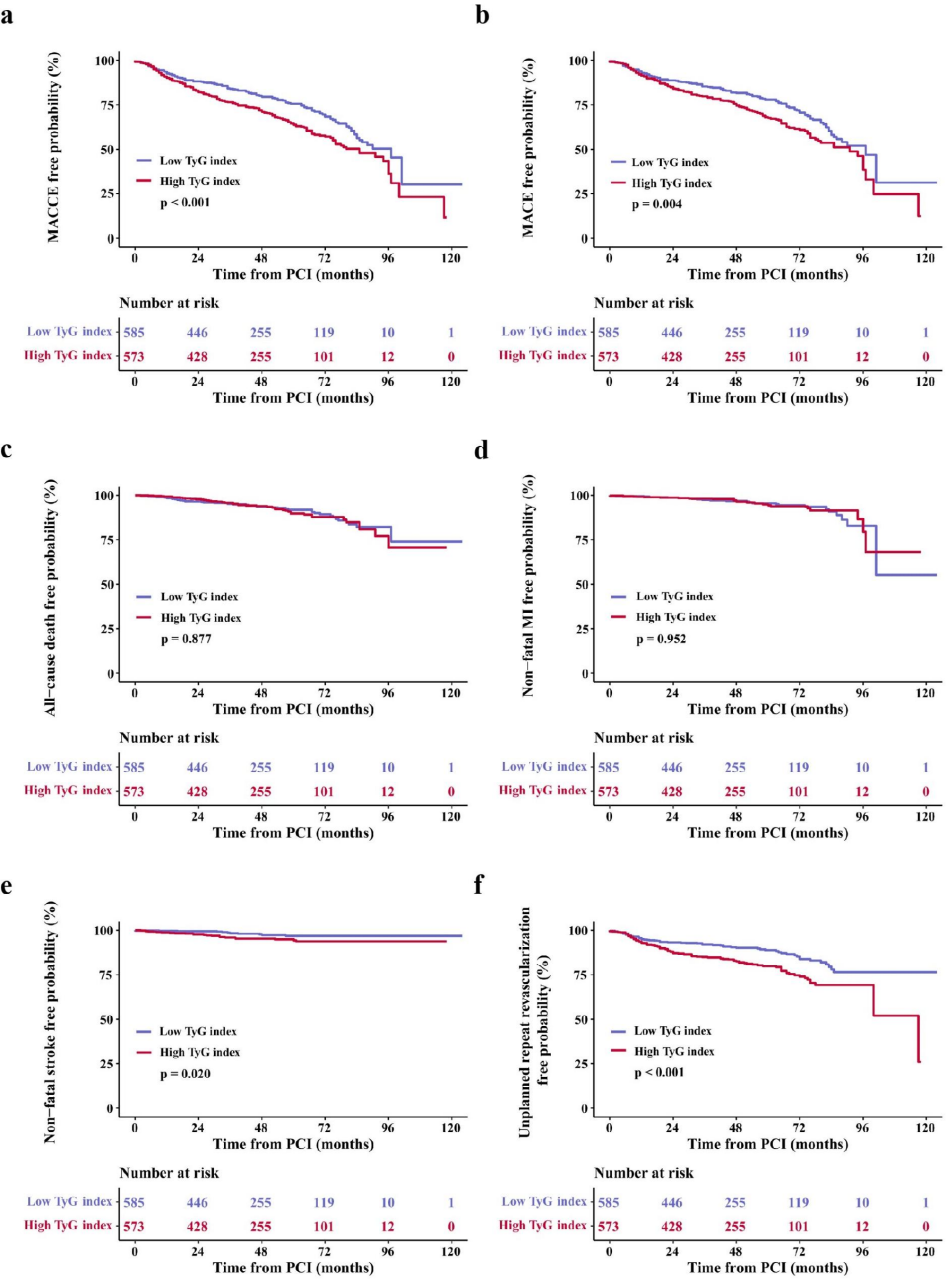
Kaplan-Meier curves for endpoint events. **a** Kaplan-Meier curves for MACCE; **b** Kaplan-Meier curves for MACE; **c** Kaplan-Meier curves for all‑cause death; **d** Kaplan-Meier curves for non‑fatal MI; **e** Kaplan-Meier curves for non-fatal stroke; **f** Kaplan-Meier curves for unplanned repeat revascularization. TyG, triglyceride-glucose; PCI, percutaneous coronary intervention; MACCE, major adverse cardiavascular and cerebrovascular events; MACE, major adverse cardiavascular events; MI myocardial infarction

Correspondingly, Kaplan-Meier survival analysis demonstrated that patients belonging to the high TyG index group experienced a noticeably elevated risk of MACCE (log-rank p < 0.001) and MACE (log-rank p = 0.004) compared with those with a lower TyG index. The difference was driven primarily by a higher incidence of unplanned repeat revascularization (log-rank p < 0.001). In addition, the rate of nonfatal stroke was higher in the high TyG index group than those in the low TyG index group (log-rank p = 0.020). There was no significant difference in the incidence of all-cause death (log-rank p = 0.877) or nonfatal MI (log-rank p = 0.952) between the two groups (Fig. 3).

Cox regression analyses were performed to evaluate the prognostic significance of the TyG index for MACCE. After adjustment with four different Cox proportional hazards models as described above, each unit increase of the TyG index was independently correlated with an elevated risk of MACCE, when the TyG index was analyzed as a continuous variable (Model 1: HR 1.21, 95% CI 1.02–1.45, p = 0.034; Model 2: HR 1.42, 95% CI 1.10–1.83, p = 0.008; Model 3: HR 1.42, 95% CI 1.09–1.83, p = 0.008; Model 4: HR 1.42, 95% CI 1.09–1.86, p = 0.009) (Table 4). When the TyG index was analyzed as a categorical variable, there was still an independent correlation between the high TyG index group and MACCE, with the low TyG index group as the reference (Model 1: HR 1.35, 95% CI 1.08–1.69, p = 0.008; Model 2: HR 1.49, 95% CI 1.14–1.94, p = 0.004; Model 3: HR 1.49, 95% CI 1.14–1.95, p = 0.003; Model 4: HR 1.53, 95% CI 1.16–2.01, p = 0.003) (Table 4). We further assessed the predictive value of the TyG index on all endpoint events with adjustment for confounding variables in Model 4. In addition to being an independent predictor of MACCE, a high TyG index was also independently associated with a high risk of MACE (HR 1.41, 95% CI 1.07–1.87, p = 0.015) and unplanned repeat revascularization (HR 1.51, 95% CI 1.04–2.19, p = 0.029), when the TyG index was analyzed as a continuous variable (Additional file 1: Table S2). This association remained when the TyG index was analyzed as a categorical variable (HR 1.49, 95% CI 1.12–1.99, p = 0.007 for MACE; HR 1.81, 95% CI 1.23–2.66, p = 0.003 for unplanned repeat revascularization) (Additional file 1: Table S2).

**Table 4:** Predictive value of TyG index for MACCE in different Cox proportional hazards models

	TyG index as a continuous variable^a^			TyG index as a categorical variable^b^		
	HR	95% CI	p value	HR	95% CI	p value
Crude model	1.28	1.09–1.51	0.003	1.45	1.17–1.79	< 0.001
Model 1	1.21	1.02–1.45	0.034	1.35	1.08–1.69	0.008
Model 2	1.42	1.10–1.83	0.008	1.49	1.14–1.94	0.004
Model 3	1.42	1.09–1.83	0.008	1.49	1.14–1.95	0.003
Model 4	1.42	1.09–1.86	0.009	1.53	1.16–2.01	0.003

Model 1: adjusted for age, male, BMI, SBP, current smoking, family history of CAD, hypertension, dyslipidemia, T2DM, prior MI, prior PCI, prior stroke, PAD, HF and CKD

Model 2: adjusted for variables in Model 1 and clinical diagnosis, Hb, hs-CRP, eGFR, TC, LDL-C, HDL-C, HbA1c and LVEF

Model 3: adjusted for variables in Model 2 and DAPT, ACEI/ARB, ARNI, antidiabetic agents and statins at discharge

Model 4: adjusted for variables in Model 3 and LM disease, multivessel disease, CTO, thrombotic disease, in-stent restenosis, target vessel selection, PTCA, number of stents, interval time from CABG to PCI and PCI success

a The HR was examined by per 1-unit increase of TyG index

b The HR was examined regarding the low TyG index group as reference

TyG, triglyceride-glucose; HR, hazard ratio; CI, confidence interval; other abbreviations as in Table 1

**Fig. 4:**
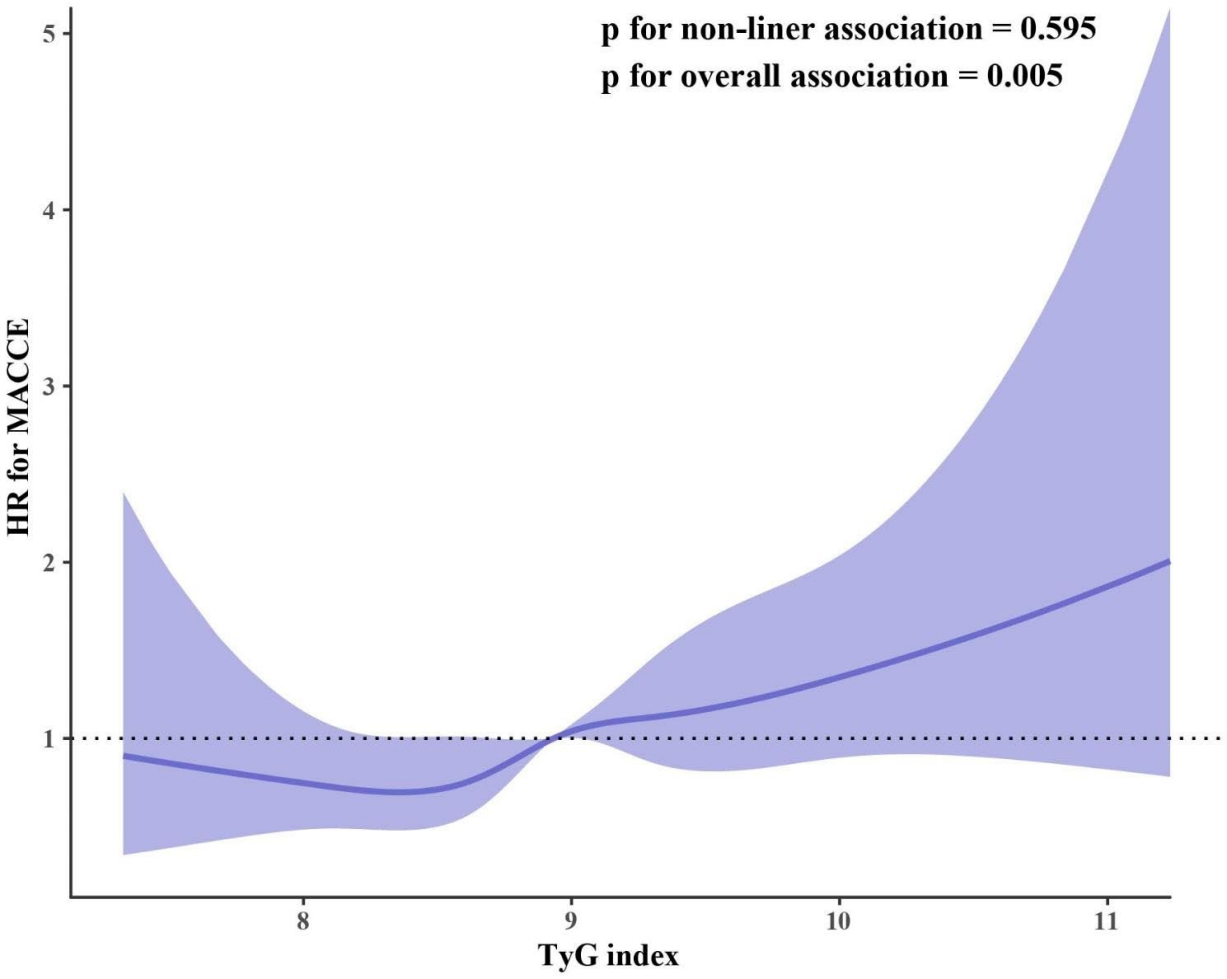
Restricted cubic splines curve for the association of TyG index with MACCE. The purple line represents the hazard ratio (HR), and the shaded area represents the 95% confidence interval (CI). The RCS analysis was performed by using Model 4 (adjusted for age, male, BMI, SBP, current smoking, family history of CAD, hypertension, dyslipidemia, T2DM, prior MI, prior PCI, prior stroke, PAD, HF, CKD, clinical diagnosis, Hb, hs-CRP, eGFR, TC, LDL-C, HDL-C, HbA1c, LVEF, DAPT, ACEI/ARB, ARNI, antidiabetic agents, statins at discharge, LM disease, multivessel disease, CTO, thrombotic disease, in-stent restenosis, target vessel selection, PTCA, number of stents, interval time from CABG to PCI, PCI success). The HR was examined by per 1-unit increase of TyG index. TyG, triglyceride-glucose; MACCE, major adverse cardiavascular and cerebrovascular events; other abbreviations as in Table 1

In addition, the RCS curve revealed a linear relationship between the TyG index and MACCE with the adjustment of Model 4 (p for non-linear = 0.595, p for overall = 0.005) (Fig. 4).

### Subgroup analysis

**Fig. 5:**
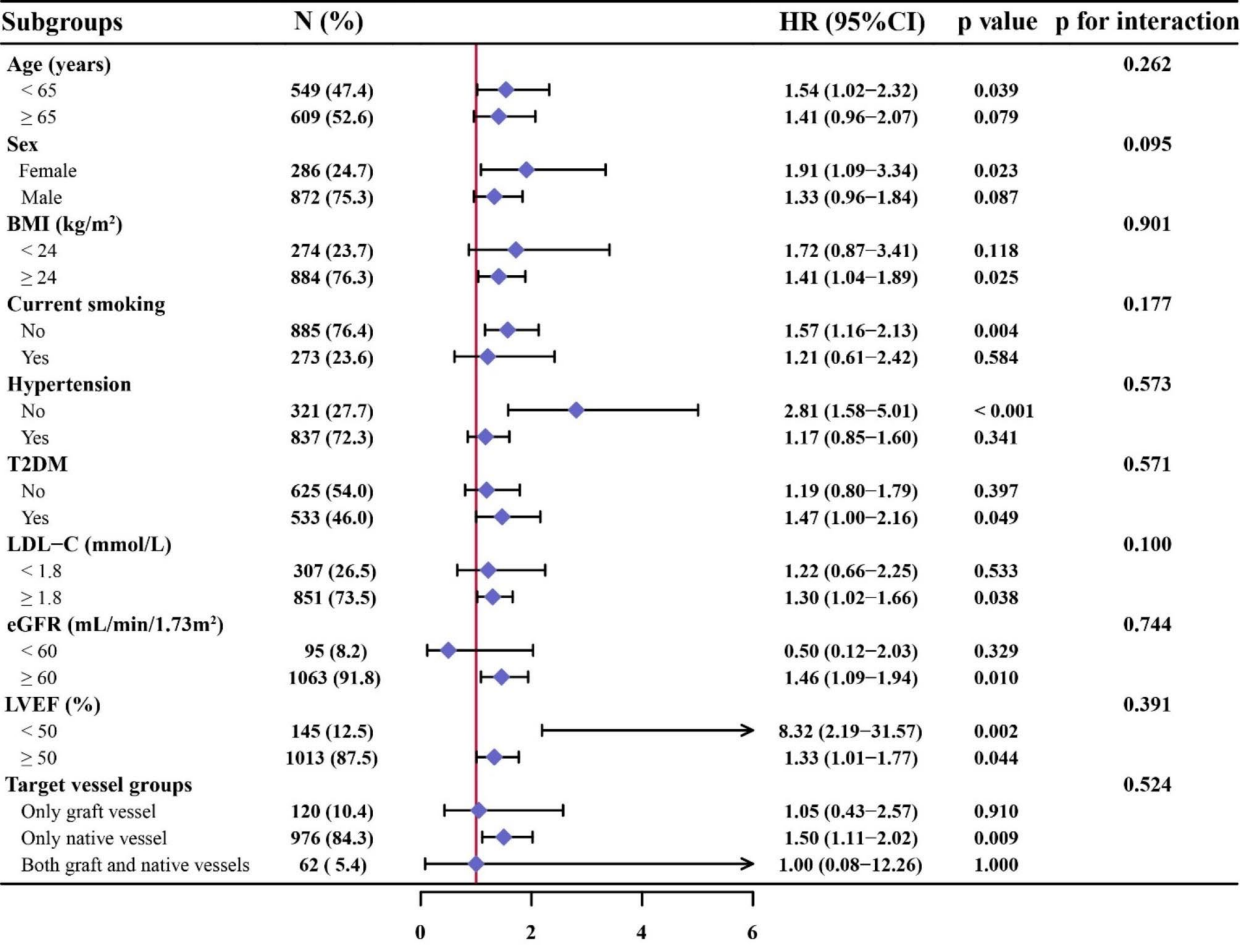
Subgroup analysis for the impact of TyG index on MACCE. The red vertical solid line represents the HR value of 1. The subgroup analysis was performed by using Model 4 (adjusted for age, male, BMI, SBP, current smoking, family history of CAD, hypertension, dyslipidemia, T2DM, prior MI, prior PCI, prior stroke, PAD, HF, CKD, clinical diagnosis, Hb, hs-CRP, eGFR, TC, LDL-C, HDL-C, HbA1c, LVEF, DAPT, ACEI/ARB, ARNI, antidiabetic agents, statins at discharge, LM disease, multivessel disease, CTO, thrombotic disease, in-stent restenosis, target vessel selection, PTCA, number of stents, interval time from CABG to PCI, PCI success). HR, hazard ratio; CI, confidence interval; BMI, body mass index; T2DM, type 2 diabetes mellitus; LDL-C, low density lipoprotein cholesterol; eGFR, estimated glomerular filtration rate; LVEF, left ventricular ejection fraction; other abbreviations as in Table 1

Subgroup analysis of the study population according to age (< 65 or ≥ 65 years), sex (female or male), BMI (< 24 or ≥ 24 kg/m^2^), current smoking (yes or no), hypertension (with or without), T2DM (with or without), LDL-C (< 1.8 or ≥ 1.8 mmol/L), eGFR (< 60 or ≥ 60 mL/min/1.73m^2^), LVEF (< 50% or ≥ 50%), and target vessel selection (only graft vessel, only native vessel, both graft and native vessels) was performed to further verify the predictive value of the TyG index for MACCE in different subgroups. As shown in Fig. 5, there was no interaction between all subgroups after adjusting for confounders with Model 4 (all p for interaction > 0.05).

### Incremental effect of the TyG index for predicting MACCE

The baseline risk model consisted of various risk factors, including age, male, BMI, SBP, current smoking, family history of CAD, hypertension, dyslipidemia, T2DM, prior MI, prior PCI, prior stroke, PAD, clinical diagnosis, eGFR, LVEF, DAPT, ACEI/ARB, antidiabetic agents, statins at discharge, LM disease, multivessel disease, CTO, in-stent restenosis, target vessel selection, number of left internal mammary artery (LIMA), number of saphenous vein graft (SVG), number of treated lesions and number of stents. The TyG index groups significantly improved the accuracy of the prediction of MACCE when added to the baseline risk model (AUC: baseline risk model, 0.618 vs. baseline risk model + TyG index groups, 0.636, p for comparison = 0.042), while the addition of FBG (p for comparison = 0.069) or TG (p for comparison = 0.773) had no such incremental effect for predicting MACCE (Fig. 2b, c and d). Furthermore, incorporating the TyG index groups into the baseline risk model produced a significant increase in AUC (p = 0.042), and significant improvements in reclassification and discrimination with an NRI of 0.257 (p < 0.001) and an IDI of 0.008 (p = 0.003) (Table 5).

**Table 5:** Incremental effect of TyG index and its components for predicting MACCE

	AUC	95%CI	p value	NRI	95%CI	p value	IDI	95%CI	p value
Baseline risk model	0.618	0.582 ~ 0.655	Ref	Ref	Ref	Ref	Ref	Ref	Ref
+TG	0.619	0.583 ~ 0.656	0.773	0.026	-0.102 ~ 0.154	0.690	0.001	-0.001 ~ 0.003	0.293
+FBG	0.631	0.595 ~ 0.668	0.069	0.191	0.064 ~ 0.317	0.003	0.007	0.002 ~ 0.012	0.009
+TyG index groups	0.636	0.600 ~ 0.672	0.042	0.257	0.129 ~ 0.386	< 0.001	0.008	0.003 ~ 0.014	0.003

Baseline risk model includes age, male, BMI, SBP, current smoking, family history of CAD, hypertension, dyslipidemia, T2DM, prior MI, prior PCI, prior stroke, PAD, clinical diagnosis, eGFR, LVEF, DAPT, ACEI/ARB, antidiabetic agents, statins at discharge, LM disease, multivessel disease, CTO, in-stent restenosis, target vessel selection, number of LIMA, number of SVG, number of treated lesions, number of stents

AUC, area under the curve; CI, confidence interval; NRI, net reclassification improvement; IDI, integrated discrimination improvement; TG, triglyceride; FBG, fasting blood glucose; TyG, triglyceride-glucose; Ref, reference; other abbreviations as in Table 1

## Discussion

The present study is the first to evaluate the predictive value of the TyG index in ACS patients with prior CABG undergoing PCI. The salient findings of our study are summarized as follows: (1) Patients with a higher TyG index exhibited a significantly greater occurrence of MACCE, MACE, nonfatal stroke, and unplanned repeat revascularization than their counterparts with a lower TyG index. (2) The TyG index was independently associated with MACCE, regardless of whether the TyG index was a continuous variable or a categorical variable. (3) Incorporating the TyG index groups into the baseline risk model resulted in an enhanced ability to predict the risk of MACCE. (4) The relationship between the TyG index and MACCE was linear, and the TyG index was associated with various cardiovascular risk factors. In conclusion, the TyG index has shown potential as a reliable prognostic indicator for evaluating long-term outcomes in ACS patients with prior CABG undergoing PCI. This is beneficial for improving the risk stratification and management of such patients.

The TyG index was a simple, convenient, economical, and reliable surrogate indicator to identify IR [10]. IR has been confirmed to have a strong correlation with the occurrence and development of CVD [8, 9]. Moreover, the TyG index has been proven in previous studies to be significantly related to arterial stiffness [13], coronary artery calcification [26], and intima-media thickness [27], all of which contribute to the incidence and development of CVD. Previous studies have shown that the TyG index is not only independently related to the incidence of CAD [28, 29], but is also an important predictor of prognosis in patients with CAD [30]. Luo et al. revealed that STEMI patients with a higher TyG index were at a greater risk of adverse CV events after PCI [22]. Similar findings were found in non-ST-segment elevation acute coronary syndrome (NSTE-ACS) patients, with or without diabetes mellitus [31, 32].

The TyG index has shown a strong risk-predictive ability in different cohorts, but almost all previous studies excluded patients with a history of CABG, so there were very few studies that evaluated the TyG index on the prediction of long-term outcomes in patients after CABG. Only two previous studies have shown that a high TyG index remained an independent risk factor for adverse CV events in T2DM patients with prior CABG after adjusting for confounders [33, 34]. However, to the best of our knowledge, no study has reported the predictive value of the TyG index in patients with prior CABG undergoing PCI. Compared with patients without prior CABG, patients with a history of CABG have higher risks of restenosis, procedural complications, and poor prognosis after PCI [35, 36]. As a result, determining a reliable predictor is extremely critical for risk stratification in such a high-risk population.

To fill this gap, we included ACS patients with prior CABG undergoing PCI, and demonstrated that a high TyG index may be a valid indicator of long-term outcomes in such a population for the first time. In the present study, there were more patients in the MACCE group who received graft vessel PCI and fewer patients who received native vessel PCI than those in the no-MACCE group, which is in line with the current recommendations and guidelines [37]. Although there was no significant difference in the selection of target vessel between the low and high TyG index groups, patients in the high TyG index group were still at a greater risk of MACCE than those in the low TyG index group. In addition to MACCE, the TyG index was also independently associated with MACE and unplanned repeat revascularization. Although the differences between the TyG index and death, MI, and stroke are not statistically significant, there is a trend towards statistical differences and we will extend the follow-up period to further validate this opinion. Moreover, we performed subgroup analysis and found that the TyG index failed to demonstrate good predictive value in the nondiabetic subgroup of patients, which may be due to the instability of patients’ blood glucose in stressful situations such as ACS.

Furthermore, the ability of the TyG index to improve the predictive performance of models has been demonstrated in previous studies [31, 32, 38, 39], but currently, there is no proof that the TyG index has an incremental effect for predicting adverse CV events in ACS patients with prior CABG undergoing PCI. Similar to previous studies, we found that the TyG index improved the ability to predict MACCE in this population. However, there was no incremental effect of adding FBG or fasting TG, possibly because patients had stricter control of blood glucose and lipids after CABG, and a large proportion of patients were routinely treated with statins or antidiabetic agents prior to admission. This not only interferes with the true levels of FBG and fasting TG, but also weakens the effects of FBG and fasting TG on prognosis. Therefore, compared with FBG or fasting TG, the TyG index might help to better identify high-risk patients with prior CABG undergoing PCI and facilitate better risk stratification and management. However, it is worth noting that the AUC values in the present study were not particularly excellent. Several factors could contribute to the predictive performance of the model. Firstly, variations in study populations, including demographics, comorbidities, and disease severity, can significantly influence the predictive performance of the model. The characteristics of our study population, such as previous history of CABG, may differ from those in previous studies, contributing to the differences in accuracy. Moreover, differences in the methodology employed, including the statistical methods used, covariates adjusted for, and duration of follow-up, can also impact the obtained results. Although the predictive performance of our model is limited, the TyG index still has potential clinical application as the number of patients with prior CABG undergoing PCI continues to grow. Further studies are needed to improve the predictive performance of the model in the future.

At present, the optimal cut-off value of the TyG index has not yet been harmonized. In our study, there was an increased risk of MACCE among patients with a TyG index of more than 8.94, suggesting that this point has some reference value, which can be used as an early warning signal for initiating lifestyle changes in patients with prior CABG undergoing PCI, as well as a reminder for clinicians to make early interventions to reduce the incidence of MACCE. Further large-scale studies are needed to identify the optimal cut-off value of the TyG index.

There has been insufficient study on the mechanism connecting the TyG index and the occurrence of MACCE. Several potential mechanisms currently proposed are based on IR. First, IR can promote the formation of atherosclerosis and plaque progression through different pathways [40]: (1) IR can induce lipid metabolism disorders, and then cause dyslipidemia [41]; (2) IR can induce disturbances in glucose metabolism, and then trigger an inflammatory response and oxidative stress, leading to vascular endothelial cell damage [42]; (3) IR can promote leukocyte adhesion to endothelial cells and impair endothelial function, further leading to plaque progression [8, 43]; (4) IR can significantly down-regulate insulin receptors and insulin receptor-mediated signaling pathways, which can promote the process of atherosclerosis [44]. Second, IR can promote thrombin synthesis and platelet aggregation, and increase the concentration of plasminogen activator inhibitor 1, which in turn causes abnormalities in the fibrinolytic system and coagulation imbalance, leading to thrombosis [45, 46]. Third, IR is also closely related to coronary microcirculation disorders, myocardial injury, and poor myocardial reperfusion, resulting in poor prognosis of patients [47]. All the above explanations could be the potential causes for patients with a higher TyG index having poor long-term outcomes.

### Study limitations

The present study has some limitations. First, it is a single-center, observational, and retrospective study, which cannot determine the causal relationship between the TyG index and MACCE, and selection bias or potential confounding factors cannot be completely ruled out. Second, the TyG index was measured only once, and the fluctuations of the TyG index for a long time were unknown, which could lead to bias in the study. Future dynamic monitoring of the long-term level of the TyG index after PCI is needed to verify this finding. Third, some patients had taken lipid-lowering agents or glucose-lowering agents before or during admission, which may have affected TG and FBG levels. Despite adjusting the use of lipid-lowering agents and glucose-lowering agents, the type, intensity, and variability of these medications were not considered, and the results may be biased. In addition, as insulin levels are not routinely measured at our center, HOMA-IR and HEC were not compared with the TyG index. As the first study to assess the predictive value of the TyG index in the population with prior CABG undergoing PCI, it still has potential clinical significance despite its limitations.

## Conclusions

In ACS patients with prior CABG undergoing PCI, an elevated TyG index is independently related to a higher risk of MACCE, and there is an incremental effect of the TyG index on the prediction of MACCE. In summary, the TyG index is beneficial to risk stratification and prognosis prediction in ACS patients with prior CABG undergoing PCI, which may help to identify the high-risk patients earlier and adopt prompt treatment strategies. Further prospective, multicenter, large sample trials are needed to determine the effect of the TyG index on long-term outcomes in ACS patients with prior CABG undergoing PCI, as well as to assess the optimal cut-off value of the TyG index.

### Electronic supplementary material

Below is the link to the electronic supplementary material.

Additional File 1: Table S1 Relationship between TyG index and cardiovascular risk factors. Table S2 Predictive value of TyG index for endpoint events in univariate and multivariate.

## Data Availability

The datasets used and/or analyzed during the current study are available from the corresponding author on reasonable request.

## Abbreviations

TyG: Triglyceride-glucose
IR: Insulin resistance
CVD: Cardiovascular disease
ACS: Acute coronary syndrome
CABG: Coronary artery bypass grafting
PCI: Percutaneous coronary intervention
MACCE: Major adverse cardiavascular and cerebrovascular events
HR: Hazard ratio
CI: Confidence interval
MACE: Major adverse cardiovascular events
AUC: Area under the curve
CAD: Coronary artery disease
LM: Left main coronary artery
CV: Cardiovascular
T2DM: Type 2 diabetes mellitus
MetS: Metabolic syndrome
HEC: Hyperinsulinemic-euglycemic clamp
HOMA-IR: Homeostasis model assessment of IR
FBG: Fasting blood glucose
TG: Triglyceride
ALT: Alanine transaminase
AST: Aspartate transaminase
ULN: Upper limit of normal
SBP: Systolic blood pressure
DBP: Diastolic blood pressure
MI: Myocardial infarction
PAD: Peripheral artery disease
HF: Heart failure
CKD: Chronic kidney disease
BMI: Body mass index
UA: Unstable angina
NSTEMI: Non‑ST‑segment elevation myocardial infarction
STEMI: ST‑segment elevation myocardial infarction
WBC: White blood cell
Hb: Hemoglobin
PLT: Platelet
hs-CRP: High-sensitivity C-reactive protein
TC: Total cholesterol
LDL-C: Low-density lipoprotein cholesterol
HDL-C: High-density lipoprotein cholesterol
HbA1c: Glycosylated hemoglobin A1c
eGFR: Estimated glomerular filtration rate
LVEF: Left ventricular ejection fraction
GRACE: Global registry of acute coronary events
SD: Standard deviation
IQR: Interquartile ranges
ROC: Receiver operating characteristic
DAPT: Dual antiplatelet therapy
ACEI/ARB: Angiotensin converting enzyme inhibitor or angiotensin receptor blocker
ARNI: Angiotensin receptor-neprilysin inhibitor
CTO: Chronic total occlusion
PTCA: Percutaneous transluminal coronary angioplasty
RCS: Restricted cubic splines
NRI: Net reclassification improvement
IDI: Integrated discrimination improvement
LAD: Left anterior descending artery
LCX: Left circumflex artery
RCA: Right coronary artery
DCB: Drug-coated balloon
BMS: Bare mental stent
DES: Drug-eluting stent
LIMA: Left internal mammary artery
SVG: Saphenous vein graft
NSTE-ACS: Non-ST-segment elevation acute coronary syndrome

## References

[1] Roth GA, Mensah GA, Johnson CO, Addolorato G, Ammirati E, Baddour LM (2020). Global Burden of Cardiovascular Diseases and Risk factors, 1990–2019: Update from the GBD 2019 study. J Am Coll Cardiol.

[2] Vaduganathan M, Mensah GA, Turco JV, Fuster V, Roth GA (2022). The Global Burden of Cardiovascular Diseases and Risk: a compass for Future Health. J Am Coll Cardiol.

[3] Writing Committee M, Lawton JS, Tamis-Holland JE, Bangalore S, Bates ER, Beckie TM (2022). 2021 ACC/AHA/SCAI Guideline for Coronary Artery revascularization: a report of the American College of Cardiology/American Heart Association Joint Committee on Clinical Practice guidelines. J Am Coll Cardiol.

[4] Chen L, Théroux P, Lespérance J, Shabani F, Thibault B, De Guise P (1996). Angiographic features of vein grafts versus ungrafted coronary arteries in patients with unstable angina and previous bypass Surgery. J Am Coll Cardiol.

[5] Goldman S, Zadina K, Moritz T, Ovitt T, Sethi G, Copeland JG (2004). Long-term patency of saphenous vein and left internal mammary artery grafts after coronary artery bypass Surgery: results from a Department of Veterans affairs Cooperative Study. J Am Coll Cardiol.

[6] Morrison DA, Sethi G, Sacks J, Henderson WG, Grover F, Sedlis S (2002). Percutaneous coronary intervention versus repeat bypass Surgery for patients with medically refractory myocardial ischemia: AWESOME randomized trial and registry experience with post-CABG patients. J Am Coll Cardiol.

[7] Reaven GM, Banting. lecture 1988. Role of insulin resistance in human disease. Diabetes 1988;37:1595 – 607. 10.2337/diab.37.12.1595 3056758

[8] Laakso M, Kuusisto J (2014). Insulin resistance and hyperglycaemia in Cardiovascular Disease development. Nat Rev Endocrinol.

[9] Ginsberg HN (2000). Insulin resistance and Cardiovascular Disease. J Clin Invest.

[10] Du T, Yuan G, Zhang M, Zhou X, Sun X, Yu X (2014). Clinical usefulness of lipid ratios, visceral adiposity indicators, and the triglycerides and glucose index as risk markers of insulin resistance. Cardiovasc Diabetol.

[11] Guerrero-Romero F, Simental-Mendia LE, Gonzalez-Ortiz M, Martinez-Abundis E, Ramos-Zavala MG, Hernandez-Gonzalez SO (2010). The product of triglycerides and glucose, a simple measure of insulin sensitivity. Comparison with the euglycemic-hyperinsulinemic clamp. J Clin Endocrinol Metab.

[12] Simental-Mendia LE, Rodriguez-Moran M, Guerrero-Romero F (2008). The product of fasting glucose and triglycerides as surrogate for identifying insulin resistance in apparently healthy subjects. Metab Syndr Relat Disord.

[13] Wang S, Shi J, Peng Y, Fang Q, Mu Q, Gu W (2021). Stronger association of triglyceride glucose index than the HOMA-IR with arterial stiffness in patients with type 2 Diabetes: a real-world single-centre study. Cardiovasc Diabetol.

[14] Park HM, Lee HS, Lee YJ, Lee JH (2021). The triglyceride-glucose index is a more powerful surrogate marker for predicting the prevalence and incidence of type 2 Diabetes Mellitus than the homeostatic model assessment of insulin resistance. Diabetes Res Clin Pract.

[15] Wang Y, Yang W, Jiang X (2021). Association between triglyceride-glucose index and Hypertension: a Meta-analysis. Front Cardiovasc Med.

[16] Park B, Lee HS, Lee YJ (2021). Triglyceride glucose (TyG) index as a predictor of incident type 2 Diabetes among nonobese adults: a 12-year longitudinal study of the Korean genome and epidemiology study cohort. Transl Res.

[17] Khan SH, Sobia F, Niazi NK, Manzoor SM, Fazal N, Ahmad F (2018). Metabolic clustering of risk factors: evaluation of triglyceride-glucose index (TyG index) for evaluation of insulin resistance. Diabetol Metab Syndr.

[18] Sajdeya O, Beran A, Mhanna M, Alharbi A, Burmeister C, Abuhelwa Z (2022). Triglyceride glucose index for the prediction of subclinical Atherosclerosis and arterial stiffness: a Meta-analysis of 37,780 individuals. Curr Probl Cardiol.

[19] Sanchez-Inigo L, Navarro-Gonzalez D, Fernandez-Montero A, Pastrana-Delgado J, Martinez JA (2016). The TyG index may predict the development of cardiovascular events. Eur J Clin Invest.

[20] Wang L, Cong HL, Zhang JX, Hu YC, Wei A, Zhang YY (2020). Triglyceride-glucose index predicts adverse cardiovascular events in patients with Diabetes and acute coronary syndrome. Cardiovasc Diabetol.

[21] Zhao Q, Cheng YJ, Xu YK, Zhao ZW, Liu C, Sun TN (2021). Comparison of various insulin resistance surrogates on prognostic prediction and stratification following percutaneous coronary intervention in patients with and without type 2 Diabetes Mellitus. Cardiovasc Diabetol.

[22] Luo E, Wang D, Yan G, Qiao Y, Liu B, Hou J (2019). High triglyceride-glucose index is associated with poor prognosis in patients with acute ST-elevation Myocardial Infarction after percutaneous coronary intervention. Cardiovasc Diabetol.

[23] Collet JP, Thiele H, Barbato E, Barthelemy O, Bauersachs J, Bhatt DL (2021). 2020 ESC guidelines for the management of acute coronary syndromes in patients presenting without persistent ST-segment elevation. Eur Heart J.

[24] Ibanez B, James S, Agewall S, Antunes MJ, Bucciarelli-Ducci C, Bueno H (2018). 2017 ESC guidelines for the management of acute Myocardial Infarction in patients presenting with ST-segment elevation: the Task Force for the management of acute Myocardial Infarction in patients presenting with ST-segment elevation of the European Society of Cardiology (ESC). Eur Heart J.

[25] Lawton JS, Tamis-Holland JE, Bangalore S, Bates ER, Beckie TM, Bischoff JM (2022). 2021 ACC/AHA/SCAI Guideline for Coronary Artery revascularization: a report of the American College of Cardiology/American Heart Association Joint Committee on Clinical Practice guidelines. J Am Coll Cardiol.

[26] Won KB, Park EJ, Han D, Lee JH, Choi SY, Chun EJ (2020). Triglyceride glucose index is an Independent predictor for the progression of coronary artery calcification in the absence of heavy coronary artery calcification at baseline. Cardiovasc Diabetol.

[27] Jia X, Zhu Y, Qi Y, Zheng R, Lin L, Hu C (2022). Association between triglyceride glucose index and carotid intima-media thickness in obese and nonobese adults. J Diabetes.

[28] Park GM, Cho YR, Won KB, Yang YJ, Park S, Ann SH (2020). Triglyceride glucose index is a useful marker for predicting subclinical coronary artery Disease in the absence of traditional risk factors. Lipids Health Dis.

[29] Liu X, Tan Z, Huang Y, Zhao H, Liu M, Yu P (2022). Relationship between the triglyceride-glucose index and risk of Cardiovascular Diseases and mortality in the general population: a systematic review and meta-analysis. Cardiovasc Diabetol.

[30] Liang S, Wang C, Zhang J, Liu Z, Bai Y, Chen Z (2023). Triglyceride-glucose index and coronary artery Disease: a systematic review and meta-analysis of risk, severity, and prognosis. Cardiovasc Diabetol.

[31] Zhao Q, Zhang TY, Cheng YJ, Ma Y, Xu YK, Yang JQ (2020). Impacts of triglyceride-glucose index on prognosis of patients with type 2 Diabetes Mellitus and non-ST-segment elevation acute coronary syndrome: results from an observational cohort study in China. Cardiovasc Diabetol.

[32] Zhao Q, Zhang T-Y, Cheng Y-J, Ma Y, Xu Y-K, Yang J-Q (2021). Triglyceride-glucose index as a surrogate marker of insulin resistance for Predicting Cardiovascular outcomes in nondiabetic patients with Non-ST-Segment elevation Acute Coronary Syndrome undergoing percutaneous coronary intervention. J Atheroscler Thromb.

[33] Chen L, Ding XH, Fan KJ, Gao MX, Yu WY, Liu HL (2022). Association between triglyceride-glucose index and 2-Year adverse Cardiovascular and cerebrovascular events in patients with type 2 Diabetes Mellitus who underwent off-pump coronary artery bypass grafting. Diabetes Metab Syndr Obes.

[34] Zhang H, Chong H, Li Z, Li K, Zhang B, Xue Y (2022). Triglyceride-glucose index in the prediction of major adverse cardiovascular events in patients with type 2 Diabetes Mellitus after coronary artery bypass Surgery: a retrospective cohort study. Front Endocrinol (Lausanne).

[35] Welsh RC, Granger CB, Westerhout CM, Blankenship JC, Holmes DR, O’Neill WW (2010). Prior coronary artery bypass graft patients with ST-segment elevation Myocardial Infarction treated with primary percutaneous coronary intervention. JACC Cardiovasc Interv.

[36] Azzalini L, Ojeda S, Karatasakis A, Maeremans J, Tanabe M, La Manna A (2018). Long-term outcomes of Percutaneous Coronary Intervention for Chronic Total Occlusion in patients who have undergone coronary artery bypass grafting vs those who have not. Can J Cardiol.

[37] Neumann FJ, Sousa-Uva M, Ahlsson A, Alfonso F, Banning AP, Benedetto U (2019). 2018 ESC/EACTS guidelines on myocardial revascularization. Eur Heart J.

[38] Xiong S, Chen Q, Chen X, Hou J, Chen Y, Long Y (2022). Adjustment of the GRACE score by the triglyceride glucose index improves the prediction of clinical outcomes in patients with acute coronary syndrome undergoing percutaneous coronary intervention. Cardiovasc Diabetol.

[39] Zhu Y, Liu K, Chen M, Liu Y, Gao A, Hu C (2021). Triglyceride-glucose index is associated with in-stent restenosis in patients with acute coronary syndrome after percutaneous coronary intervention with drug-eluting stents. Cardiovasc Diabetol.

[40] Semenkovich CF (2006). Insulin resistance and Atherosclerosis. J Clin Invest.

[41] Ormazabal V, Nair S, Elfeky O, Aguayo C, Salomon C, Zuñiga FA (2018). Association between insulin resistance and the development of Cardiovascular Disease. Cardiovasc Diabetol.

[42] Beverly JK, Budoff MJ, Atherosclerosis (2020). Pathophysiology of insulin resistance, hyperglycemia, hyperlipidemia, and inflammation. J Diabetes.

[43] Wu G, Meininger CJ (2009). Nitric oxide and vascular insulin resistance. BioFactors.

[44] Bornfeldt KE, Tabas I (2011). Insulin resistance, hyperglycemia, and Atherosclerosis. Cell Metab.

[45] Stegenga ME, van der Crabben SN, Levi M, de Vos AF, Tanck MW, Sauerwein HP (2006). Hyperglycemia stimulates coagulation, whereas hyperinsulinemia impairs fibrinolysis in healthy humans. Diabetes.

[46] Undas A, Wiek I, Stêpien E, Zmudka K, Tracz W (2008). Hyperglycemia is associated with enhanced thrombin formation, platelet activation, and fibrin clot resistance to lysis in patients with acute coronary syndrome. Diabetes Care.

[47] Trifunovic D, Stankovic S, Sobic-Saranovic D, Marinkovic J, Petrovic M, Orlic D (2014). Acute insulin resistance in ST-segment elevation Myocardial Infarction in non-diabetic patients is associated with incomplete myocardial reperfusion and impaired coronary microcirculatory function. Cardiovasc Diabetol.

