# Supplementary material for: Triglyceride-glucose index is associated with poor prognosis in acute coronary syndrome patients with prior coronary artery bypass grafting undergoing percutaneous coronary intervention
Source: Cardiovasc Diabetol. 2023 Oct 27;22:286. doi: 10.1186/s12933-023-02029-6 (PMC10612342; doi:10.1186/s12933-023-02029-6)
Supplement: Supplementary file 1 — Additional File 1: Table S1 Relationship between TyG index and cardiovascular risk factors. Table S2 Predictive value of TyG index for endpoint events in univariate and multivariate. [file 12933_2023_2029_MOESM1_ESM.docx]

**Additional file 1**

**Table S1 Relationship between TyG index and cardiovascular risk factors**

| **Variable** | **Correlation coefficient** | **p value** |
| --- | --- | --- |
| Age | -0.108 | <0.001 |
| Male | -0.113 | <0.001 |
| BMI | 0.152 | <0.001 |
| Current smoking | 0.003 | 0.928 |
| Family history of CAD | 0.044 | 0.136 |
| Hypertension | 0.039 | 0.186 |
| Dyslipidemia | -0.005 | 0.854 |
| T2DM | 0.307 | <0.001 |
| hs-CRP | 0.188 | <0.001 |
| eGFR | -0.057 | 0.052 |
| TC | 0.230 | <0.001 |
| LDL-C | 0.186 | <0.001 |
| HDL-C | -0.241 | <0.001 |
| HbA1c | 0.424 | <0.001 |
| LVEF | -0.005 | 0.858 |
| GRACE risk score | 0.020 | 0.494 |
| Target vessel selection | -0.051 | 0.085 |

All abbreviations as in Table 1.

**Table S2 Predictive value of TyG index for endpoint events in univariate and multivariate analysis**

|  | **Univariate analysis** | | | **Multivariate analysis^a^** | | |
| --- | --- | --- | --- | --- | --- | --- |
|  | **HR** | **95% CI** | **p value** | **HR** | **95% CI** | **p value** |
| **TyG index as a continuous** **variable^b^** |  |  |  |  |  |  |
| MACCE | 1.28 | 1.09-1.51 | 0.003 | 1.42 | 1.09-1.86 | 0.009 |
| MACE | 1.25 | 1.05-1.48 | 0.012 | 1.41 | 1.07-1.87 | 0.015 |
| All-cause death | 1.10 | 0.79-1.52 | 0.574 | 1.82 | 1.05-3.17 | 0.033 |
| Non-fatal MI | 1.00 | 0.65-1.54 | 0.990 | 0.95 | 0.45-1.97 | 0.882 |
| Non-fatal stroke | 1.63 | 0.99-2.67 | 0.053 | 1.56 | 0.62-3.89 | 0.343 |
| Unplanned revascularization | 1.41 | 1.12-1.77 | 0.003 | 1.51 | 1.04-2.19 | 0.029 |
| **TyG index as a categorical variable^c^** |  |  |  |  |  |  |
| MACCE | 1.45 | 1.17-1.79 | <0.001 | 1.53 | 1.16-2.01 | 0.003 |
| MACE | 1.38 | 1.11-1.73 | 0.004 | 1.49 | 1.12-1.99 | 0.007 |
| All-cause death | 1.03 | 0.68-1.57 | 0.876 | 1.34 | 0.76-2.37 | 0.311 |
| Non-fatal MI | 0.98 | 0.56-1.71 | 0.953 | 1.11 | 0.50-2.47 | 0.799 |
| Non-fatal stroke | 2.28 | 1.12-4.66 | 0.023 | 2.16 | 0.83-5.57 | 0.112 |
| Unplanned revascularization | 1.71 | 1.31-2.40 | <0.001 | 1.81 | 1.23-2.66 | 0.003 |

a The multivariate analysis was performed by using Model 4 (adjusted for age, male, BMI, SBP, current smoking, family history of CAD, hypertension, dyslipidemia, T2DM, prior MI, prior PCI, prior stroke, PAD, HF, CKD, clinical diagnosis, Hb, hs-CRP, eGFR, TC, LDL-C, HDL-C, HbA1c, LVEF, DAPT, ACEI/ARB, ARNI, antidiabetic agents, statins at discharge, LM disease, multivessel disease, CTO, thrombotic disease, in-stent restenosis, target vessel selection, PTCA, number of stents, interval time from CABG to PCI, PCI success).

b The HR was examined by per 1-unit increase of TyG index.

c The HR was examined regarding the low TyG index group as reference.

HR, hazard ratio; CI, confidence interval; TyG, triglyceride-glucose; MACCE, major adverse cardiavascular and cerebrovascular events; MACE, major adverse cardiavascular events; MI, myocardial infarction; other abbreviations as in Table 1.
